# *Coriandrum sativum* L.: A Review on Ethnopharmacology, Phytochemistry, and Cardiovascular Benefits

**DOI:** 10.3390/molecules27010209

**Published:** 2021-12-30

**Authors:** Nisa Najibah Mahleyuddin, Said Moshawih, Long Chiau Ming, Hanis Hanum Zulkifly, Nurolaini Kifli, Mei Jun Loy, Md. Moklesur Rahman Sarker, Yaser Mohammed Al-Worafi, Bey Hing Goh, Shobna Thuraisingam, Hui Poh Goh

**Affiliations:** 1 PAP Rashidah Sa’adatul Bolkiah Institute of Health Sciences, Universiti Brunei Darussalam, Seri Begawan BE1410, Brunei; nisanjbh@gmail.com (N.N.M.); saeedmomo@hotmail.com (S.M.); long.ming@ubd.edu.bn (L.C.M.); nurolaini.kifli@ubd.edu.bn (N.K.); 2 Faculty of Pharmacy, Universiti Teknologi MARA, Shah Alam 42300, Malaysia; hanish2984@uitm.edu.my; 3 Faculty of Engineering, Universiti Teknologi Malaysia, Johor Bahru 81310, Malaysia; junmeiloy@gmail.com; 4 Department of Pharmacy, State University of Bangladesh, Dhaka 1205, Bangladesh; moklesur2002@yahoo.com; 5 College of Medical Sciences, Azal University for Human Development, Amran P.O. Box 447, Yemen; yworafi@yahoo.com; 6 College of Pharmacy, University of Science and Technology of Fujairah, Fujairah P.O. Box 2202, United Arab Emirates; 7 Biofunctional Molecule Exploratory (BMEX) Research Group, School of Pharmacy, Monash University Malaysia, Subang Jaya 47500, Malaysia; goh.bey.hing@monash.edu; 8 College of Pharmaceutical Sciences, Zhejiang University, Hangzhou 310058, China; 9 Department of Chemistry, Faculty of Science, Universiti Putra Malaysia, Kuala Lumpur 43400, Malaysia; shobnasima@gmail.com

**Keywords:** *Coriandrum sativum*, cardiovascular, coriander, antihypertensive, hypolipidemic, cardioprotective, functional food, ethnopharmacology

## Abstract

*Coriandrum sativum* (*C. sativum*), belonging to the Apiaceae (Umbelliferae) family, is widely recognized for its uses in culinary and traditional medicine. *C. sativum* contains various phytochemicals such as polyphenols, vitamins, and many phytosterols, which account for its properties including anticancer, anti-inflammatory, antidiabetic, and analgesic effects. The cardiovascular benefits of *C. sativum* have not been summarized before, hence this review aims to further evaluate and discuss its effectiveness in cardiovascular diseases, according to the recent literature. An electronic search for literature was carried out using the following databases: PubMed, Scopus, Google Scholar, preprint platforms, and the Cochrane Database of Systematic Reviews. Articles were gathered from the inception of the database until August 2021. Moreover, the traditional uses and phytochemistry of coriander were surveyed in the original resources and summarized. As a result, most of the studies that cover cardiovascular benefits and fulfilled the eligibility criteria were in vivo, while only a few were in vitro and clinical studies. In conclusion, *C. sativum* can be deemed a functional food due to its wide range of cardiovascular benefits such as antihypertensive, anti-atherogenic, antiarrhythmic, hypolipidemic as well as cardioprotective effects.

## 1. Introduction

*Coriandrum sativum* Linn. (*C. sativum*) or coriander, is recognized for its wide range of uses in culinary as well as traditional medicine in a variety of conditions [1]. Different chemical compounds have been identified in each part of the plant including roots, leaves, fruits, and seeds, which account for its broad spectrum of uses [2]. To name a few, such compounds include gallic acid, thymol, and bornyl acetate, which are expected to exert anticancer, anti-inflammatory, and autonomic relaxation induction effects, respectively [3,4,5]. Linalool, a terpene alcohol found in coriander, has been reported to be the main constituent that is responsible for some therapeutic values of coriander as it possesses neuroprotective, anxiolytic, anticonvulsant, and analgesic effects [6,7,8,9].

It is believed that every part of the plant possesses different nutritional and medicinal values; thus, it was traditionally consumed in diverse areas. Notably, coriander was used in India for relieving gastrointestinal discomfort, respiratory, and urinary complaints; additionally, in some areas of Pakistan, the whole plant of coriander has folk medicinal uses to treat flatulence, dysentery, diarrhea, and vomiting [10,11]. On the other hand, with its distinctive scent and flavor, coriander is often added to food in the culinary industry as a seasoning and a preservative agent; it can be used in the form of leaves and seeds, ground, or as a whole [12]. Furthermore, the powdered fruit of *C. sativum* has been used as a flavoring agent to mask the taste of some foods such as fish, meat, and baking recipes [13].

According to the World Health Organization [14] (WHO, 2019), the major cause of mortality worldwide is cardiovascular diseases (CVDs). CVDs are a group of heart and blood vessel disorders including coronary and peripheral artery diseases, rheumatic, cerebrovascular, and congenital heart disease, in addition to deep vein thrombosis and pulmonary embolism [15]. Despite possessing various health benefits which have been reported in many research and review papers [16,17,18], the cardioprotective effects of coriander have never been summarized in terms of the anti-atherogenic, antihypertensive, antiarrhythmic, and hypolipidemic effects [19]. Phytochemicals present in *C. sativum*, such as flavonoids, phenolic acids, phytosterols, and terpenes, have significant potential in cardiovascular health and have demonstrated an angiotensin-converting enzyme (ACE)-inhibiting potency, cardioprotective, antihyperlipidemic, and cardiometabolic disorder-inhibiting properties [20,21,22,23]. Therefore, the phytochemical constituents of *C. sativum* were reviewed and summarized in light of their presence in their respective extracts and connected to the biological activities shown. Moreover, this review also aims to further evaluate, summarize, and discuss the cardiovascular effects of coriander extracts in all contexts of in vitro, in vivo, and clinical studies that were performed on this herb.

### 1.1. Botanical Description and Taxonomy

**Figure 1:**
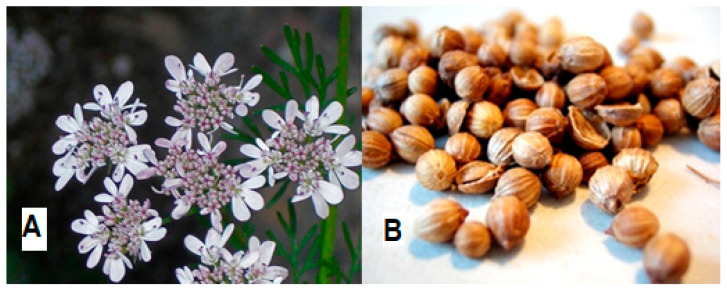
(**A**) *C. sativum* flowers (by Andrey Zharkikh). (**B**) *C. sativum* half and whole seeds (by ZoyaChubby). From North Carolina Extension Gardner: Plant toolbox. (https://plants.ces.ncsu.edu/plants/coriandrum-sativum/, accessed on 20 December 2021).

**Figure 2:**
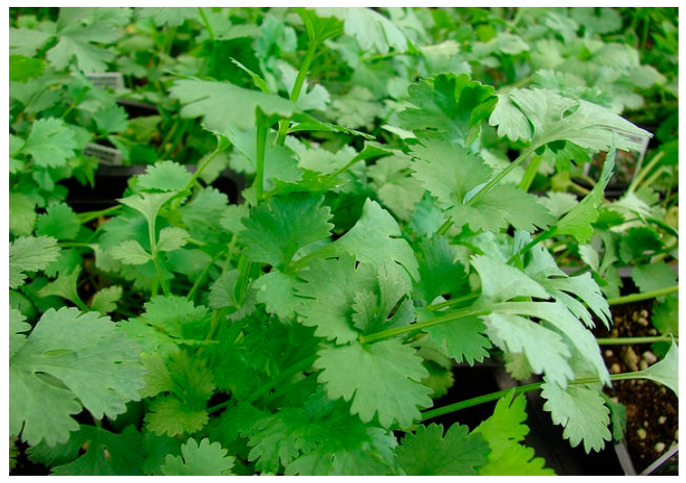
The leaves of *Coriandrum sativum* (by Forest and Kim Starr). From North Carolina Extension Gardner: Plant toolbox. (https://plants.ces.ncsu.edu/plants/coriandrum-sativum/, accessed on 20 December 2021).

*C. sativum* belongs to the Apiaceae (Umbelliferae) family, which is herbaceous and grows annually, with a height of 20–70 cm (Table 1). *C. sativum* is known as “coriander” or “Chinese parsley” in English; “cilantro” in Spanish; “kusthumbari” or “dhanya” in Sanskrit; “dhane” in Bengali; “pak chee” in Thailand; and “Yánsuī”, “Yán qiàn”, “Hú suī”, or “Xiāngcài” in Chinese [1,2,24]. It is thought to have originated in the regions of the Middle East and the Mediterranean, where its growth may have broadened to China, Europe, India, Africa, and Asia; nevertheless, several authors have considered coriander as a weed in cereals, and its origin is still not clear [25]. The leaves are green with a variable lanceolate shape and glabrous surfaces, while the flowers are white or pink in umbels with asymmetrical shapes (Figure 1 and Figure 2) [2,18]. Meanwhile, the seeds are dry schizocarps with two mericarps with oval-shaped globules. Furthermore, the stems of *C. sativum* are pale green with hollow branches and a glabrous surface [24].

### 1.2. Ethnomedicinal Uses

*C. sativum* was used as one of the earliest spices by humans. Traditionally, *C. sativum* seeds were consumed to relieve pain, rheumatoid arthritis, and inflammation [26], whereas the decoction of coriander was believed to treat mouth ulcers and eye redness [27]. The seeds have been prescribed to relieve gastrointestinal disorders such as flatulence, diarrhea, indigestion, and nausea [28]. Coriander is believed to exert these actions by stimulating the liver to increase the secretion of bile and other digestive enzymes which escalate the action of the digestive system, hence shortening the time of food passage through the gastrointestinal tract [29]. In countries such as Saudi Arabia, Jordan, and Morocco, coriander was also known to lower blood glucose levels [30,31,32], have antimicrobial properties against food-borne pathogens, such as Salmonella, in addition to aphrodisiac and analgesic power [33]. Furthermore, coriander has been used traditionally in Turkey and India to relieve indigestion [34]; increase water excretion; and prevent seizures, anxiety, and sleeplessness [35,36]. Moreover, it has been documented of *C. sativum* in Morocco being used traditionally in the treatment of diabetes, indigestion, flatulence, insomnia, renal disorders, loss of appetite, and as a diuretic [37]. Detailed traditional uses of *C. sativum* are presented in Table 2.

### 1.3. Phytochemistry

**Figure 3:**
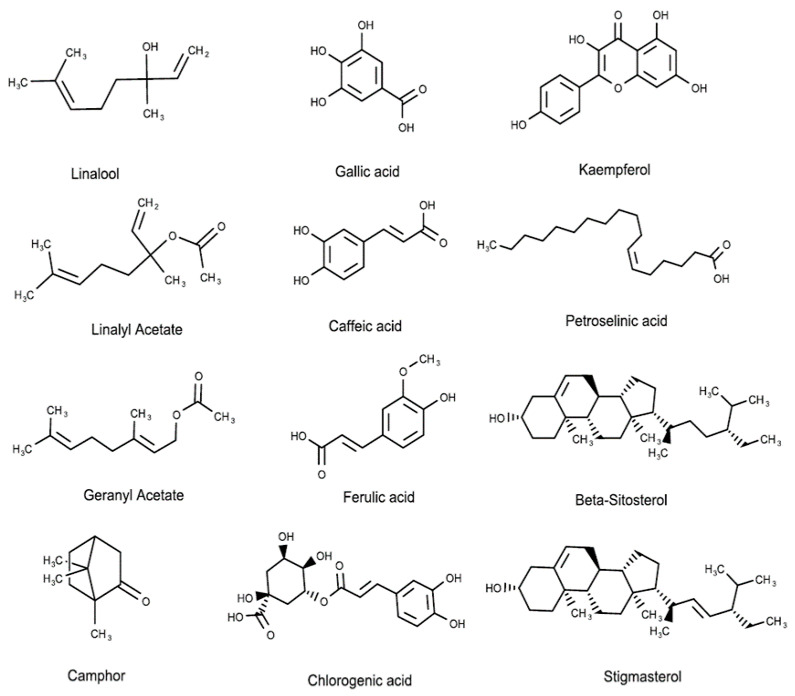
Phytochemical molecular structures extracted from *C. sativum*, such as terpenes, phenolic acids, flavonoids, fatty acids, and phytosterols.

Recent studies revealed that different kinds of alkaloids, essential oils, fatty acids, flavonoids, phenolics, reducing sugars, sterols, tannins, and terpenoids were extracted from *C. sativum* [16,44]. In particular, the leaves were reported to have an abundant concentration of folates, ascorbic acid, gallic acid, caffeic acid, ferulic acid, and chlorogenic acid. Additionally, the investigation of the water-soluble components of *C. sativum* seeds showed the presence of 33 compounds, including monoterpenoid, monoterpenoid glycosides and glucosides, and aromatic compound glycosides such as norcarotenoid glucoside [45]. In the vegetative part of the *C. sativum*, different phenolics and flavonoids were detected in significantly high concentrations, such as quercetin diverse glycosides (405.36–3296.16 mg/kg), kaempferol 3-*O*-rutinoside (320.86 mg/kg), in addition to ferulic acid glucoside and *p*-coumaroylquinic acid [46]. Another study of the polyphenolic contents of coriander grass showed that a 40% ethanol extract contains many flavonoids (0.13% to 10.71%), coumarins (1.4% to 6.83%), and phenolcarboxylic acids (7.24% to 13.51%) [47]. Anthocyanin was also characterized in coriander leaves, and the concentration was found to be influenced by salicylic acid, nitrogen, phosphorus, potassium, and zinc fertilizers [48]. Figure 3 and Table 3 present the different phytochemical structure classes of *C. sativum.*

These constituents of *C. sativum* were extracted by different methods, including solvent extraction such as n-hexane, water, and methanol, in addition to supercritical gases, microwave-assisted extraction, sonication, and hydrodistillation [20,49,50,51,52,53,54]. Essential oils (EOs) and fatty oils were the most significant components in the fruit, with a content of 0.03–2.6% and 9.9–27.7%, respectively [55]. Different EO chemotypes were detected in coriander seeds, including ketones, aliphatic aldehydes, aliphatic hydrocarbon, aliphatic alcohols, esters, monoterpene hydrocarbons, monoterpene oxides, monoterpene alcohols, monoterpene esters, and sesquiterpenes, with linalool as the main monoterpene alcohol extracted in high quantities [56]. The highest percentage of EO that has been yielded from coriander seeds was cultivated in Tunisia with a linalool content of 87.54% [57], while 73.1%, 40.9–79.9%, 37.65%, and 69.60% of linalool was extracted from *C. sativum* from Algeria, Iran, Bangladesh, and Pakistan, respectively [58,59,60].

EOs are obtainable by extracting different parts of the plant using hydrodistillation extraction [10], subcritical water, Soxhlet apparatus [61], solvent extraction, steam distillation, and supercritical CO_2_ methods [62]. However, the EO content is variable among different parts of the plant, which could be due to the different origins of cultivated varieties, climate and geographical conditions [10,63], the area and season of cultivation, the degree of plant maturation, and genotypes of the species. In particular, 2-dodecenal was found as the main volatile EO in the root and stalk, while 1-ethenyl-cyclododecanol was the highest volatile component in the leaves of *C. sativum* [64]. Furthermore, different percentages, components, and immunotoxicity were identified in oils extracted from the leaves than the stems’ EO extracts of the Korean *C. sativum* [65]. In addition, the essential oil yields and efficiencies of commercial coriander fruits from different countries were extracted and found to be different according to the geographical area [66]. The relationship between some environmental conditions in Argentina and the essential oil composition of two coriander landraces (European and Argentinean) has been studied [67]. It has also been found that genotypic and phenotypic variations contributed to the variety of essential oils’ concentrations, in addition to the interaction from other environmental conditions, such as fertilizers, weediness, and soil degradation. Likewise, the phenolic contents of the two *C. sativum* varieties, *vulgare* and *microcarpum,* were found to be similar but with different concentrations of the main phenolic compounds, namely quercetin-3-b-d glucoside and quercetin-3-*O*-glucuronide [68]. On top of that, the average EO yields for *vulgare* fruits (0.1% to 0.5%) are lower than that of the *microcarpum* (0.8–2.1%) [16]. Details of the total phytochemical constituents of *C. sativum* are presented in Table 3.

**Table 3:** Main phytochemical constituents of *C. sativum* classified according to their chemical class, including the plant part that was extracted and the extraction method.

Phytochemical Components	Chemical Class	Plant Part	Extraction Solvent/Method	Reference
Ferulic acid, Gallic acid, and Caffeic acid	Phenolic acids	Above-ground parts	Ether, ethyl acetate, butanol, and 2-ethyl acetate extracts	[47]
Salicylic acid	Benzoic acid derivative			
Esculetin, Esculin, Scopoletin 4-Hydroxycoumarin, Umbelliferone, and Dicoumarin	Coumarins			
Hyperoside, rutin, hesperidin, vicenin, diosmin, luteolin, apigenin, orientine, dihydroquercetin, catechin, and arbutin	Flavonoids			
β-carotene and total carotenoids	Carotenoids	Leaves at mature and young plant stage, fresh and dry seeds	Ice-cold acetone was then partitioned against petroleum ether.	[69]
α-, β-, γ- δ- tocopherols, and α-, γ-tocotrienols	Tocols	Whole fruit, pericarp, and seeds	Extracted with *n*-hexane	[70]
Petroselinic acid, linoleic acid, palmitic acid, and oleic acid	Fatty acids		Boiled in water, then grounded using a mixture of chloroform/methanol/hexane and finally separated by thin-layer chromatography	
Stigmasterol, β-sitosterol, δ-stigmasterol	Sterols	Seed and pericarp of coriander fruit	Extracted with *n*-hexane in a Soxhlet apparatus	[71]
Linalool, camphor, and geraniol	Essential oils		Hydrodistillation followed by extraction with 2-methylbutane	

**Figure 4:**
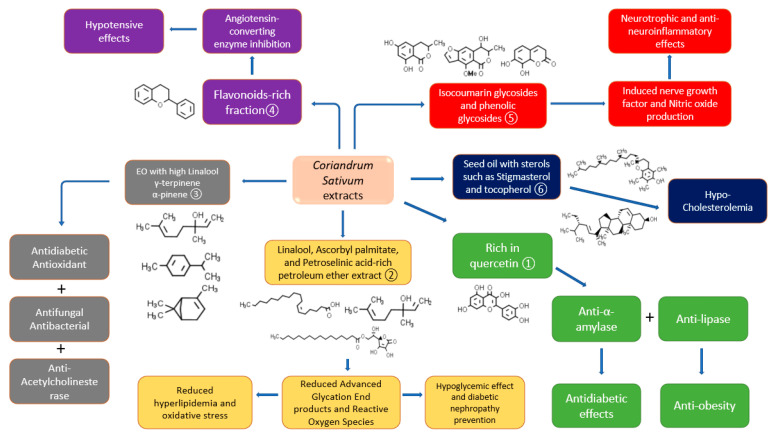
The role of *C. sativum* phytochemicals in some of its biological activities from different studies ① Suttisansanee, Thiyajai, Chalermchaiwat, Wongwathanarat, Pruesapan, Charoenkiatkul and Temviriyanukul [72], ② Kajal and Singh [73], ③ Hajlaoui, Arraouadi, Noumi, Aouadi, Adnan, Khan, Kadri and Snoussi [75], ④ Hajlaoui, Arraouadi, Noumi, Aouadi, Adnan, Khan, Kadri and Snoussi [75], ⑤ Cha, Yoon, Kim, Kim and Lee [76], and ⑥ Ramadan, et al. [77].

Different studies involved the characterization of *C. sativum* phytochemicals in the extracts that were employed in bioactivity determination. However, very few studies involved the bioactivity-guided isolation of a specific compound that is responsible for that pharmacological action. Quercetin-rich aqueous ethanolic extract was found beneficial in α-amylase and α-glucosidase inhibition and thus has a potential antidiabetic effect [72]. The extract also contained some other flavonoid compounds such as vanillic acid, caffeic acid, and p-coumaric acid, whereas the total phenolic content was 2.68 ± 0.07 mg GAE/g DW. Lipase inhibition activity for 1 mg/mL of aqueous ethanol was 55.76 ± 1.40% which indicates a significantly strong anti-obesity property. In the same study, the angiotensin-converting enzyme was inhibited by coriander extracts to 70.66 ± 2.34% which shows that the polyphenolic-rich extract can be beneficial for hypertension in vitro. Moreover, the diabetic nephropathy prevention and hypolipidemic activities were illustrated in the petroleum ether extract of *C. sativum* seeds that are rich in linalool, ascorbyl palmitate, and petroselinic acid [73]. The saturation of hexokinase enzymes due to diabetes leads to the formation of advanced glycation end products (AGEs), which when interacting with their receptors cause vascular aging and renal damage [74]. From the above-mentioned study, it was found that linalool in addition to other terpenes and fatty acids has the potential to bind with the RAGE receptor and subsequently block AGEs’ damaging action. In a different study, the Eos’ major components of *C. sativum* were linalool, γ-terpinene, and α-pinene with prominent activities against diabetes, microbial infections, and acetylcholinesterase enzyme [75]. Other activities such as hypotensive [20] and neuroprotective ones [76] were detected in flavonoids- and isocoumarin glycosides-containing fractions of *C. sativum,* respectively. The isocoumarin glycosides cilantroside A and B, in addition to the phenolic glycosides daphnin and benzyl-*O*-β-d-glucoside, have been found to stimulate nerve growth factor as the main neurotrophic factor which is related to nerve growth, maintenance, and neuronal survival. Moreover, the aglycones of the isocoumarins showed anti-inflammatory effects in addition to more significant neurotrophic properties than their glycosides. Figure 4 indicates the details on studies performed on *C. sativum* with the characterization or isolation of the phytochemicals in the respective extract/fraction.

## 2. Results

From previous studies, *C. sativum* has demonstrated its cardioprotective efficacies (refer to Graphical Abstract). These include its effect as an antioxidant, antihypertensive, anti-atherogenic, antiarrhythmic, as well as the improvement of other factors that may lead to CVDs, such as increased lipid profile and cardiac biomarkers or enzymes. From the PRISMA flow diagram, Figure 3 demonstrates the search strategy that has been used and its results; a total of 3008 studies were screened and 13 were excluded from this review as duplicate records before the screening. A total of 2977 studies were further excluded for not meeting the determined inclusion criteria, thus leaving only 18 studies to be retrieved and included in this review. Twelve in vivo, two in vitro, and two clinical studies in addition to two mixed in vivo and in vitro studies were retrieved. The results of the studies included in this review, plant parts extracted, extraction method, dose employed, phytochemicals characterized, and isolation method are summarized in Table 4.

## 3. Discussion

### 3.1. Hypolipidemic Activity

Crude *C. sativum* extract has been shown to lower the levels of triacylglycerol (TCA) and total cholesterol (TC), indicating its potential in decreasing blood lipid profile in rats [77,79,80,82,83,86,87,88,90]. Bioactive sterols, which contain mono- and polyunsaturated fatty acids, such as stigmasterols and sitosterols, and polar lipids, are present in coriander extract in high amounts [77]. In addition, the presence of antioxidants, such as tocopherols and phenolics, protects the unsaturated fatty acids from peroxidation [91]. Saturated fatty acids likely elevate the low-density lipoprotein cholesterol (LDL-C) levels, while unsaturated fatty acids, including monounsaturated and polyunsaturated fatty acids (PUFA), reduce LDL-C levels [92]. The higher intake of PUFA-containing oils in food helps in incorporating more unsaturated fatty acids in plasma lipoproteins, which improves their particle size and subclass distribution more than when it involves a higher content of cholesterol particles due to the fact that the fatty esters such as PUFA are bigger in size [93]. Additionally, cholesterol as a rigid fat will be incorporated more as a cell membrane component instead of circulating in blood since the fluidity of unsaturated acids in membranes must be balanced with rigid fats [94]. Moreover, phytochemical components such as flavonoids and polyphenols can also be responsible for the hypolipidemic property of coriander, whereby the flavonoids are responsible for reducing FAS proteins which are involved in energy metabolism. The inhibition of the expression of FAS in the liver by stimulating AMPK activity in hepatocyte cells via the liver kinase B1 pathway may thereby reduce fatty acid synthesis in the liver and subsequently, fat accumulation [95].

Another potential hypolipidemic mechanism for coriander seeds proposed by Chithra and Leelamma [88] may involve a significant increase in the 3-hydroxy-3-methylglutaryl coenzyme A reductase (HMG-CoA) activity which is a key enzyme in the biosynthesis of cholesterol. Since liver cholesterol was significantly reduced in rats administered coriander, it was most likely because the rate of its degradation to bile acids was more than its rate of synthesis. Other than that, Sharma, Sharma, Jasuja and Joshi [79] found an increase in the excretion of cholesterol and phospholipids in the fecal matter of rabbits that were administered *C. sativum* seed extract. Generally, plant sterols and stanols reduce intestinal absorption of cholesterol, increase neutral fecal sterol excretion, and prevent liver cholesterol accumulation; however, they did not cause liver X receptor target gene induction, such as *Abcg5*, *Abcg8,* or *Npc1l1.* Thus, phytosterol-induced cholesterol absorption reduction was independent of Abcg5/8 Transporter [96].

In contrast, the reduction of TC levels was attributed to the amount of fibers added to the experimental diet of hypertriglyceridemic rats [88,97], which further increases plasma lecithin cholesterol acyltransferase (LCAT) activity, amplifies the synthesis of hepatic bile acids, thus increasing the cholesterol degradation in the fecal excretion of bile acids and cholesterol, and decreases the LDL liver production. Coriander seed oil is rich in sterols, predominantly stigmasterol and β-sitosterol, which work preemptively on inhibiting the absorption of dietary cholesterol [88,98]. The slight difference between stigmasterol and cholesterol structures allows the former to be incorporated in intestinal micelle to displace cholesterol and subsequently reduces the latter absorption. Furthermore, phytosterols, especially stigmasterol, increase the enzyme activity of HMG-CoA reductase, which suppresses cholesterol absorption and enhances hepatic bile acids synthesis and thus, increases the degradation of cholesterol [99].

### 3.2. Antioxidant and Anti-Atherogenic Properties

Antioxidants play an important role in the promotion of cardiovascular health since oxidative stress that inflicts the myocardium can be associated with the incidence of atherosclerosis, which increases the risk of coronary artery disease [100,101]. *C. sativum* has been demonstrated in many studies to possess a pronounced antioxidant activity, and this is mainly due to the activity of polyphenols, vitamins, and sterol constituents of coriander [78,89,90].

In particular, the antioxidant mechanism that is correlated with the ability of *C. sativum* phytochemicals is to inhibit scavenger receptor (SRB1) expression, which in turn decreases the number of foam cell formations and subsequently reduces the atherogenic plaques [79,89]. Furthermore, the atherosclerosis mechanism involves the accumulation of LDL-C within the walls of arteries, which then develop into a cholesterol plaque. Described as an inflammatory disease, atherosclerosis involves the oxidation processes by reactive oxygen species and lipoxygenases, along with enzymatic changes that occur to the entrapped LDL-C and extracellular matrix (ECM). Those changes increase the generation of adhesion molecules and chemokines from endothelial cells that boost the adhesiveness of monocytes on the cell wall, which also facilitates the infiltration of inflammatory cells into the subendothelial spaces [102,103]. This results in the recruitment of macrophages to scavenge this oxidized LDL (Ox-LDL) via scavenger receptor (SRB1), which is subsequently converted into lipid-laden foam cells [104]. Eventually, these cells undergo apoptotic death in which the apoptotic bodies mimic a fatty streak [105]. Furthermore, this increases the recruitment of macrophages having a similar fate, whereby the buildup of the by-products promotes atherosclerosis [105]. Therefore, coriander extract lowers the development of Ox-LDL in a dose-dependent manner as it has demonstrated the reduction in the number of foam cells due to its potential to prevent the expression of SRB1 [79,89].

Another potential antioxidant mechanism is associated with the high levels of polyphenol in the leaf extract of *C. sativum* [106,107], which acts as a free radical scavenger, thus preventing oxidative damage to the myocardial tissues [89]. Free radicals are highly reactive; for instance, hydroxyl radicals can cause damage to membrane phospholipids, DNA, and proteins, in which the former would result in the formation of peroxyl radicals [77]. Thus, when there are insufficient natural antioxidant components in the body, such as superoxide dismutase (SOD) enzyme and glutathione peroxidase (GSH-px), an oxidative chain reaction may occur [78], resulting in tissue damage [108]. However, it has been reported that this strong antioxidant potential of coriander may also be due to the synergistic effect of the combination of different antioxidant compounds, such as tocopherols and sterols [77].

Plants with green leaves such as coriander produce volatile oils, usually composed of (E)-2-Alkenals, emitted when wounds and stress are applied to them as a defense mechanism. N-pentane extract of *C. sativum* accounts for 70% of the components of its essential oil to this kind of green leaf volatiles [109]. It has been suggested that those alkenals attach to Keap1 proteins through electrophilic modification by the α, β-unsaturated aldehyde group, resulting in Nrf2 activation. This activation leads to detoxification and ROS neutralization in different in vivo and in vitro assays [110]. Furthermore, umbelliferone is a coumarin derivative that was extracted from *C. sativum* using methanol [111] and is also found to increase the expression of Nrf2 in addition to CREB, SIRT1, FOXO-3, PPAR-γ genes, and proteins. SIRT1 upregulation plays a key role in regulating oxidative defense mechanisms and DNA repair [112] and can be considered one of the pathways in which coriander illustrates its antioxidant and anti-atherogenic effects.

Furthermore, a study by Dhyani, Parveen, Siddiqi, Hussain and Fahim [87] demonstrated the ability of *C. sativum* seed extract to improve the cardiac hemodynamic parameters, namely systolic blood pressure (SBP), diastolic blood pressure (DBP), heart rate (HR), and mean arterial pressure (MAP) in the experimental rats. The reflective response in the case of myocardial ischemic injury is illustrated by a decline in the MAP and an increase in the HR and cardiac contractility, whereby the latter happens due to the activation of baroreceptors, eventually leading to vasoconstriction and hence enhancing the imbalance between the oxygen supply and demand in the cardiac tissues [87]. The coriander extract is expected, as illustrated in the above-mentioned studies, to improve these parameters, and ultimately, the event of MI can be prevented. A cardioprotective effect was also demonstrated for the polyphenol-rich extract of *C. sativum* due to its antioxidant potential that is associated with preventing myocardial infarction due to myofibrillar injury after the isoproterenol-induction of cardiac damage in male Wister rats [49]. The elevation of the reactive oxygen species is associated with myocardial damage, causing necrosis, apoptosis, or autophagy due to the swelling of mitochondria, which is attributed to the calcium being diverted to it after disruption in its handling between the sarcoplasmic reticulum and myofilament [113].

In addition to the mechanisms above, the histology of the aorta was also studied to determine the effect of *C. sativum* extract on cholesterol deposition in rabbits, showing almost normal histology whereby the aorta and the size of the lumen were restored to almost the normal state, thus proving the anti-atherogenic potential of coriander. In contrast to that, the histological observation of the aorta of cholesterol-fed animals without the extract treatment exhibited atheromatous plaque as compared to the normal-fed group. The lipids deposited in the atherosclerotic lesions are mostly derived from LDL, which can be oxidized by pro-oxidants, resulting in plaques characterized by lipid-laden foam cells within the innermost and middle layers of the aorta [79]. The atherogenic index serves as a crucial prognostic indicator for CVDs such as atherosclerosis [114], which represents the ratio between cholesterol and high-density lipoprotein (HDL) [115]. Increased risk of myocardial infarction is denoted by the atherogenic index of more than five [116]. Moreover, a significant decrease in this ratio by *C. sativum* extract has been reported in a previous study by Sharma, Sharma, Jasuja and Joshi [79] and Aissaoui, Zizi, Israili and Lyoussi [83], indicating another potential of coriander as a functional food to decrease the risk of CVDs.

### 3.3. Antihypertensive Potential

Another potential cardioprotective effect of *C. sativum* is its antihypertensive property [20,51]. This property is attributed to the flavonoid-rich leaves of *C. sativum*, particularly involving the angiotensin-converting enzyme (ACE) inhibition mechanism that has been illustrated in vitro by Hussain, Jahan, Rahman, Sultana and Jamil [20], whereby its IC_50_ value was 28.91 μg/mL, which is comparable to other plants possessing the same property as the IC_50_ values of plant extracts that exhibit this enzyme inhibition ranges from 16–310 μg/mL [117,118,119]. Flavonoids that have been identified in coriander leaves include quercetin, rutin, apigenin, and luteolin [120,121,122], which have also been proven to show hypotensive effects individually and in crude extracts in diverse mechanisms [123,124,125,126]. The potential mechanism in the management of hypertension through the inhibition of ACE involves the control of the production of nitric oxide (NO) by the regulatory mechanism of the renin-angiotensin-aldosterone system (RAAS) with ACE inhibition as a fundamental regulator of blood pressure [20]. NO is an essential vasodilator in the regulation of blood pressure. ACE binds with the substrate in addition to the zinc ion in the complex, resulting in the polarization of the carbonyl group, thus promoting a nucleophilic attack, which contributes to an elevation in blood pressure [20]. Therefore, some flavonoids, as well as the free hydroxyl groups of flavonoids, inhibit ACE action by chelation with the zinc ion on the active site of ACE [127], which might be due to the formation of hydrogen bridges with amino acids nearby this active site [128,129,130]. Hence, this demonstrates the cardioprotective effect of coriander extract by the action of this class of polyphenols.

Furthermore, the investigation of the antihypertensive effect of the crude extract of *C. sativum* in anesthetized rats was shown to induce the relaxation of arterial contractions, thus lowering blood pressure [50]. It has been reported that such an effect occurs due to the combination of the cholinergic and calcium channel blocking effects of coriander bioactive compounds [131]. Additionally, the diuretic effect of *C. sativum* contributes to this antihypertensive activity by increasing the loss of electrolytes in the urine output [132]. Therefore, the findings from those studies potentiate the assumption of the instrumental use of coriander extract in hypertension as a functional food.

### 3.4. Antiarrhythmic Activity

Arrhythmia is one of the CVDs, which can be defined as irregular patterns of heart rate or rhythm that could be either tachycardia or bradycardia [133]. The antiarrhythmic activity of *C. sativum* seed extract has been evaluated in a study by Rehman, Jahan, Khalil ul, Khan and Zafar [51] that resulted in the reduction of pulse rate as well as the normalization of electrocardiogram (ECG) patterns and cardiac biomarker levels, which include lactate dehydrogenase (LDH), creatine kinase-MB fraction (CK-MB), aspartate transaminase (AST), and alanine transaminase (ALT). Rehman, Jahan, Khalil ul, Khan and Zafar [51] found that the anti-tachycardia efficiency of *C. sativum* was comparable with the beta-blocker drug propranolol, but less for the anti-bradycardia effect than that of atropine, whereby the presence of polyphenolic compounds may be responsible for this action. Polyphenols bind with beta-adrenergic receptors for the regulation of heart rate, as well as having a negative chronotropic effect which may slow down myocytes’ action potential, thus possibly deterring arrhythmias [107].

## 4. Materials and Methods

**Figure 5:**
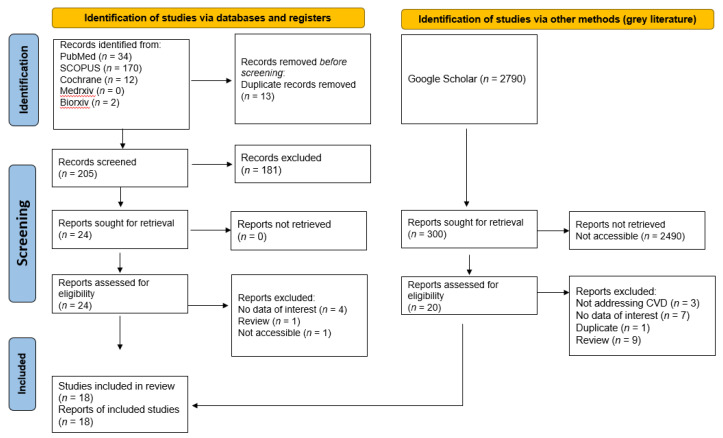
PRISMA Flow Diagram.

Electronic databases that were used for this review were PubMed, Scopus, Google Scholar, preprint platforms, and the Cochrane Database of Systematic Reviews. The articles were gathered from the inception of the database until August 2021. The strategies used for the electronic search are shown in Figure 5 (PRISMA Flow diagram).

The terms used to conduct the search included: “*Coriandrum sativum*”, coriander, cilantro, cardiovascular, hypertensive, blood pressure, atherosclerosis, myocardial, cardiology, cardiac, heart, hyperlipidemia, cholesterol, health benefit, benefit, uses, clinical effect, clinical use, medicine, ethnomedicine, phytotherapy, and ethnobotany. These terms were used in combination with the Boolean operators “AND” and “OR”, and the search strategy can be found in Supplementary Table S1. The search terms for Preprint Platform were restricted to “*Coriandrum sativum*”, coriander, and cilantro to limit the search results for the topic of interest.

The eligibility of studies to be included in this review was assessed according to the following inclusion and exclusion criteria:

Inclusion criteria:

1. Articles including research articles, guidelines, monographs, technical papers, conference proceedings;
2. Studies that are reported in the English language only,
3. Treatment of cardiovascular diseases;
4. Human studies, in vivo animal model, and in vitro laboratory studies;
5. There was no limitation imposed on the year of publication of the studies.

Exclusion criteria: Studies that assess other benefits of coriander that do not relate to cardiovascular health, reviews, letters, case studies, conference papers, opinions, reports, or editorial papers.

Screening of publications on studies relevant to coriander that examine its cardioprotective activity was conducted. Relevant literature was retrieved and reviewed in case they were related to the pharmacological properties and therapeutic actions of *C. sativum*.

## 5. Conclusions

Coriander has been widely used over the past centuries for both culinary and traditional purposes. Although linalool has been identified in *C. sativum* extract as the main component in the extracted oil, many flavonoids, phenolic acids, and phytosterols were found to be the major groups of phytochemicals that are expected to be accountable for the cardioprotective effects of coriander. In this review, the efficacy of coriander in CVDs has been discussed and evaluated according to the previous studies and reports, in which *C. sativum* is shown to exhibit hypolipidemic, antioxidant, antiatherogenic, antihypertensive, and antiarrhythmic properties in a dose-dependent manner. The included studies in this systematic review shed the light on the cardioprotective ability of coriander; however, more in vitro studies are needed to elucidate the mechanism of action of its extracts in cardiovascular diseases. Clinical trials are also warranted for fresh and extracted *C. sativum* to confirm the results presented in this review in an endeavor to consider it as a functional food and nutraceutical agent that can be consumed for therapeutic and nutritive purposes.

## Figures and Tables

**Table 1:** The taxonomical classification of *C. sativum* is as follows.

Scientific Name:	*Coriandrum sativum*
Common names:	Coriander, Chinese Parsley, cilantro, kusthumbari, dhanya, dhane, pak chee, yuan sui, hu sui
Kingdom:	Plantae
Subkingdom:	Tracheobionta
Superdivision:	Spermatophyta
Division:	Magnoliophyta
Class:	Magnoliopsida
Subclass:	Rosidae
Order:	Apiales
Family:	Apiaceae/Umbelliferae
Genus:	*Coriandrum* L.
Species:	*Coriandrum sativum* L.

**Table 2:** Traditional uses of *C. sativum* in different countries.

Traditional Uses	Area	Plant Parts Used	Reference
Rheumatoid arthritis, inflammation, and joint pain	India	Seeds/seeds aqueous extract	[26,38,39]
For measles, diabetes, aerophagy, gastroenteritis	China	The whole plant parts	[40]
Antiviral and neuro-energizer	Pakistani herbal drugs (Intellan)	Aerial parts of the plant	
Some liver diseases	-	Aqueous extract of the roasted seeds	
Carminative, diuretic, dyspeptic complaints, loss of appetite, convulsion, insomnia, and anxiety and in medical purposes	Iranian traditional medicine	Powdered seeds or dry extract	[38]
Diaphoretic, diuretic, carminative, and stimulant activity	Iranian traditional medicine	The whole plant parts	[37,41]
Diuretic and for some renal diseases	Morocco	Oral administration of plant parts	[42]
Mouth ulcer and eye redness	-	leaves decoction	[27]
Grounded as an ingredient of curry powder and gingerbread, also a component of liquesces and spirits. Aromatic ingredient of tobacco and perfumes. In Unani medicine to quench thirst and for melancholia.	India	Seeds and aqueous infusion of leaves	[39]
Stimulant and carminative; stomachic, antibilious, digestive stimulant	India	Leaves	[29]
Lower blood glucose levels	Saudi Arabia, Jordan, Morocco	Fruits, decoction of leaves and seeds	[30,31,32]
Aphrodisiac, analgesic, antimicrobial properties	-	The volatile oil	[33]
Appetizer, Digestive, Carminative	Turkey	Infusion of the seeds	[34]
For anxiety, sedative and muscle relaxant effect	-	The aqueous extract	[35,36]
Treats Influenza, bad breath, unpleasant odor from genitalia	Traditional Chinese Medicine	Seeds	[43]
Against worm and to treat rheumatism	The European pharmacopeia	Fruits	
Stimulates gastric secretion, treats gastric ulcers and mouth infections	Asian region	Essential oils	

**Table 4:** Cardiovascular potential of *C. sativum* from previous studies with details about the plant part used, extraction method, dosage em-ployed, phaytochemicals characterized, and isolation method.

Plant Part	Extraction Method	Dosage Employed	Phytochemicals Characterized	Isolation Method	Experimental Subject	Cardiovascular Effects (Outcome)	Reference
Leaves and stem	Ethanol extraction	200 mg/kg	Phenolics, flavonoids	Evaporation	Wistar albino rats	Antioxidant, Hypolipidemic Normoglycemic	Ananthan, et al. [78]
Seeds	Methanol extraction	100 mg/kg, 200 mg/kg, 300 mg/kg	N/D	Reduced pressure	Adult male Wistar rats	Decrease cardiac damage that causes myocardial infarction	Patel, Desai, Gandhi, Devkar and Ramachandran [49]
Seeds	Soxhlet extraction	250 mg/kg	N/D	Reduced pressure	Male albino rabbits	Hypolipidemic	Sharma, et al. [79]
Market-procured coriander powder suspended in water	-	1 g/kg	N/D	-	Male Wistar rats	Hypolipidemic	Lal, et al. [80]
Seed powder	-	2 g per day	N/D	-	Human individuals	Lower elevated blood pressure, Hypocholesterolemic	Zeb, et al. [81]
Market-procured seeds, powdered	-	5 g per day	N/D	-	Type 2 diabetic patients	Hypolipidemic, antioxidant	Rajeshwari, et al. [82]
Seeds	Aqueous extraction	200 mg/kg	N/D	-	*Meriones shawi* rats	Hypolipidemic Normo-glycemic Cardioprotective	Aissaoui, et al. [83]
Seeds	Hydrodistillation	IC_50_: 34.8 ± 2.3 μg/mL	Linalool	Hydrodistillation	In vitro study	Antihypertensive, Antioxidant	Chaudhary, et al. [84]
Seeds	Maceration in methanol	183 mg/kg	N/D	-	Rats	Cardioprotective	Afsheen, et al. [85]
Seeds	Homogenized seeds	10% of diet	N/D	-	Sprague-Dawley rats	Hypolipidemic	Dhanapakiam, et al. [86]
Seeds	Aqueous extraction	1 g/kg	N/D	-	Wistar albino rats	Cardioprotective, Hypolipidemic, Antioxidant, Improved left ventricle functions	Dhyani, et al. [87]
Leaves	Soxhlet extraction with multiple solvents	IC_50_: 28.91 ± 13.42 μg/mL	Pinocembrin, Apigenin, pseudobaptigenin, galangin-5-methyl ether, quercetin, baicalein, kaempferol, pinobanksin-glycosides, rutin, isorhamnetin, daidzein, luteolin, pectolinarigenin	LC-ESI-MS/MS	In vitro study	Antihypertensive	Hussain, Jahan, Rahman, Sultana and Jamil [20]
Seeds	Solvent extraction	Addition of 100 g to diet	Stigmasterol, Lanosterol, β-Sitosterol, D^5^Avenasterol, Sitostanol, D^7^stigmastenol, D7 Avenasterol, Tocopherols	Gas-liquid chromatography	Male albino rats	Hypocholesterolemic effect	Ramadan, Amer and Awad [77]
Seeds	Homogenized seeds	10% of diet	N/D	-	Female albino rats	Hypolipidemic	Chithra and Leelamma [88]
Seeds	Water extraction	300 mg/kg	N/D	-	Male albino rats	Antiarrhythmic	Rehman, Jahan, Khalil ul, Khan and Zafar [51]
Seeds	Maceration with Aqueous/Methanol	200 mg/kg	N/D	-	In vitro and in vivo study on Sprague-Dawley rats	Antioxidant, Hypocholesterolemic Anti-atherogenic	Patel, et al. [89]
Fruit	Maceration with Aqueous/Methanol	1–30 mg/mL as hypotensive 1–10 mg/kg as diuretic	N/D	Organic fractionation of the crude extract	In vivo and in vitro study	Anti-hypertensive, Diuretic	Jabeen, Bashir, Lyoussi and Gilani [50]
Leaves	Maceration with Methanol	100 mg/kg	N/D	-	Rabbits	Hypolipidemic	Kousar, et al. [90]

## Supplementary Materials

The following supporting information can be downloaded at: Table S1: Table of search terms combination and strategy.
