# Supplementary material for: Coriandrum sativum L.: A Review on Ethnopharmacology, Phytochemistry, and Cardiovascular Benefits
Source: Molecules. 2021 Dec 30;27(1):209. doi: 10.3390/molecules27010209 (PMC8747064; doi:10.3390/molecules27010209)
Supplement: Supplementary file 1 [file molecules-27-00209-s001.zip › molecules-1505147-supplementary.pdf]

Supplementary Table S1: Table of search terms combination and strategy

Database	Search term combination and strategy
Pubmed	#1 "coriandrum sativum" OR coriander OR cilantro<br>#2 Cardiovasc* OR hypertens* OR "blood pressure" OR atherosclero* OR myocardial OR cardiology OR cardiac OR heart OR hyperlipid* OR cholesterol<br>#3 "health benefit" OR benefit OR uses OR "clinical effect" OR "clinical use" OR medicin* OR ethnomedicine OR phytotherapy OR ethno?botany<br>#4 #1 AND #2 AND #3
SCOPUS	#1 "coriandrum sativum" OR coriander OR cilantro<br>#2 Cardiovasc* OR hypertens* OR "blood pressure" OR atherosclero* OR myocardial OR cardiology OR cardiac OR heart OR hyperlipid* OR cholesterol<br>#3 "health benefit" OR benefit OR uses OR "clinical effect" OR "clinical use" OR medicin* OR ethnomedicine OR phytotherapy OR ethno?botany<br>#4 #1 AND #2 AND #3
Google Scholar	("coriandrum sativum" OR coriander OR cilantro) AND (Cardiovasc* OR hypertens* OR "blood pressure" OR atherosclero* OR myocardial OR cardiology OR cardiac OR heart OR hyperlipid* OR cholesterol) AND ("health benefit" OR benefit OR uses OR "clinical effect" OR "clinical use" OR medicin* OR ethnomedicine OR phytotherapy OR ethno?botany)
Preprint Platform<br>- Medrxiv<br>- Biorxiv	"coriandrum sativum" OR coriander OR cilantro
Cochrane Central Register of Controlled Trials	#1 "coriandrum sativum" OR coriander OR cilantro<br>#2 Cardiovasc* OR hypertens* OR "blood pressure" OR atherosclero* OR myocardial OR cardiology OR cardiac OR heart OR hyperlipid* OR cholesterol<br>#3 "health benefit" OR benefit OR uses OR "clinical effect" OR "clinical use" OR medicin* OR ethnomedicine OR phytotherapy OR ethno?botany<br>#4 #1 AND #2 AND #3
